# Monitoring and Landscape Dynamic Analysis of Alpine Wetland Area Based on Multiple Algorithms: A Case Study of Zoige Plateau

**DOI:** 10.3390/s20247315

**Published:** 2020-12-19

**Authors:** Wenlong Li, Pengfei Xue, Chenli Liu, Hepiao Yan, Gaofeng Zhu, Yapeng Cao

**Affiliations:** 1 State Key Laboratory of Grassland Agro-Ecosystems, College of Pastoral Agriculture Science and Technology, Lanzhou University, Lanzhou 730020, China; wllee@lzu.edu.cn (W.L.); liuchl18@lzu.edu.cn (C.L.); yanhp19@lzu.edu.cn (H.Y.); caoyp18@lzu.edu.cn (Y.C.); 2 Key Laboratory of Grassland Livestock Industry Innovation, Ministry of Agriculture, Lanzhou University, Lanzhou 730020, China; 3 Key Laboratory of Western China’s Environmental Systems (Ministry of Education), College of Earth and Environmental Sciences, Lanzhou University, Lanzhou 730020, China; zhugf@lzu.edu.cn

**Keywords:** wetland, random forest, Google Earth Engine (GEE), Zoige Plateau, landscape analysis

## Abstract

As an important part of the wetland ecosystem, alpine wetland is not only one of the most important ecological water conservation areas in the Qinghai–Tibet Plateau region, but is also an effective regulator of the local climate. In this study, using three machine learning algorithms to extract wetland, we employ the landscape ecological index to quantitatively analyze the evolution of landscape patterns and grey correlation to analyze the driving factors of Zoige wetland landscape pattern change from 1995 to 2020. The following results were obtained. (1) The random forest algorithm (RF) performs best when dealing with high-dimensional data, and the accuracy of the decision tree algorithm (DT) is better. The performance of the RF and DT is better than that of the support vector machine algorithm. (2) The alpine wetland in the study area was degraded from 1995 to 2015, whereas wetland area began to increase after 2015. (3) The results of landscape analysis show the decrease in wetland area from 1995 to 2005 was mainly due to the fragmentation of larger patches into many small patches and loss of the original small patches, while the 2005 to 2015 decrease was caused by the loss of many middle patches and the decrease in large patches from the edge to the middle. The 2015 to 2020 increase is due to an increase in the number of smaller patches and recovery of original wetland area. (4) The grey correlation degree further shows that precipitation and evaporation are the main factors leading to the change in the landscape pattern of Zoige alpine wetland. The results are of great significance to the long-term monitoring of the Zoige wetland ecosystem.

## 1. Introduction

Wetland provides many important environmental services, including flood storage, drought control, regional climate regulation, erosion control, degradation of environmental pollutants, biodiversity, and habitat [1,2,3,4,5,6]. As a unique component of the wetland system, alpine wetland on the Qinghai–Tibet Plateau is an important natural resource for the headwaters of the Yangtze and Yellow Rivers [7,8], the first and second largest rivers in China, respectively, and is one of the most important living environments for local Tibetans. However, serious degradation of wetland has been threatening the sustainable development of the regional economy and ecosystem. Wetland degradation is further weakening water conservation in the Yangtze and Yellow River headwaters and has been a major cause of the Yellow River’s flow reduction and the seasonal drying in recent decades [9,10]. Thus, there is an urgent need for advanced technologies and methods to monitor local alpine wetland [11] in order to provide a scientific basis for the protection and management of regional wetland resources.

Remote sensing technology has made wetland monitoring more rapid, efficient, and large-scale [12,13]. The methods of wetland data extraction and classification based on remote sensing mainly include artificial visual interpretation and computer-automated classification. Visual interpretation has high requirements for the interpreter’s interpretation experience, and consumes a lot of time and energy, so it is not suitable as an independent classification method. Computer-automated algorithms can be divided into supervised classification and unsupervised classification [14,15,16]. Many researchers have proved that the supervised classification algorithm based on sample training is much better than unsupervised classification [14,15,16,17,18]. The random forest (RF), support vector machine (SVM), and decision tree (DT) supervision classification algorithms are the three most popular algorithms [17,18,19] in wetland classification at present, and they each have their own advantages. Considering RF and SVM are more often used on the Google Earth Engine (GEE) platform [20,21,22,23,24], while DT on the traditional platform [25,26], we select the classification method and platform according to most research preferences and further search for the best classification scheme for alpine wetland area.

Wetland degradation can be quantitatively analyzed through the evolution of landscape patterns [27,28,29]. Landscape indexes can highly concentrate landscape pattern information and reflect some characteristics of its structural composition and spatial allocation. It can be used to quantitatively describe and monitor the changes in landscape spatial structure over time. The landscape pattern index can be divided into patch level index, patch type level index and landscape level index. The patch level index is the basis for calculating other types of landscape indexes. The patch type level index and landscape level index are of great significance for describing and understanding the characteristics of different patch types and the overall landscape pattern in the landscape. The landscape pattern has been changing, which is the result of various factors inside and outside the landscape on different spatio-temporal scales. Identifying the driving factors of landscape pattern change and exploring its driving mechanism is a necessary condition for in-depth understanding of surface landscape evolution [29] and controlling the process of landscape pattern change. Grey correlation degree is a method [30,31,32,33] to measure the strength, size, and order of the relationship between things or system factors. In this study, it can analyze the driving factors of wetland landscape pattern change and explain and sort the factors leading to pattern change in order to reveal the change characteristics and rules of the landscape change’s driving factors.

Given the background above, this study combined remote sensing images, the landscape ecological index and grey correlation to solve three main questions: (1) what is the best classification scheme for alpine wetland areas? (2) How does the landscape pattern in Zoige wetland change? (3) What are the main factors affecting the change in Zoige landscape pattern?

## 2. Materials and Methods

### 2.1. Study Area

**Figure 1:**
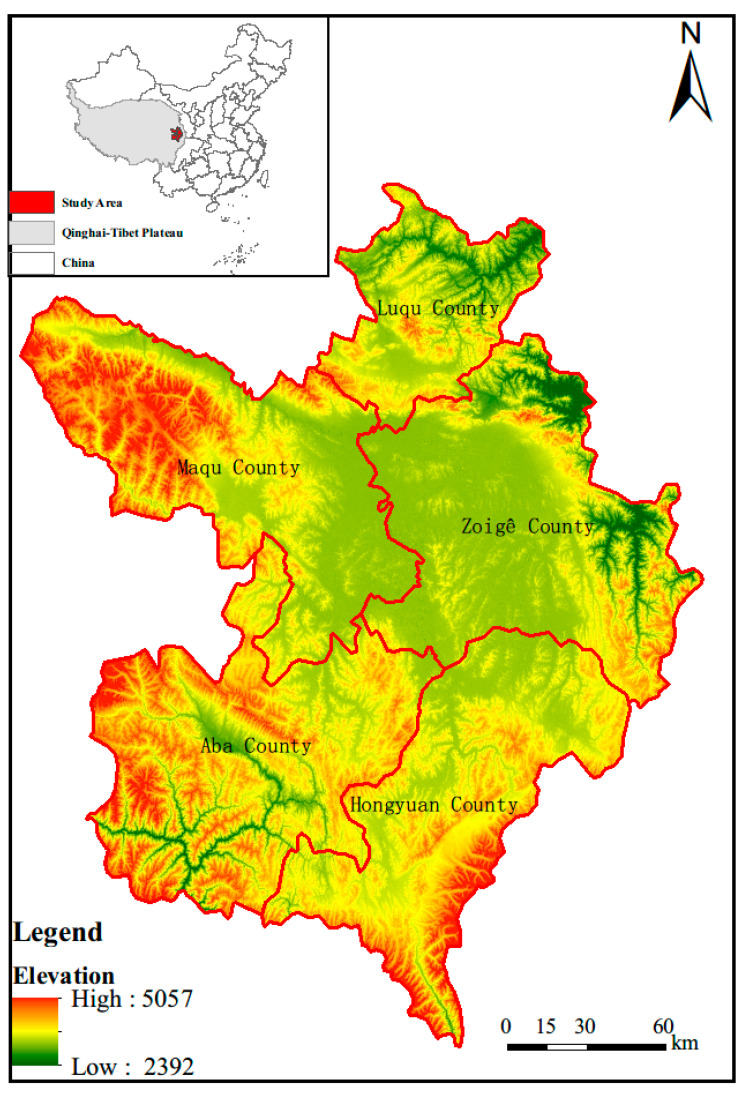
Location of Zoige Plateau (ZP).

The Zoige Plateau (ZP) (101°36′~103°25′ E, 32°20′~34°06′ N) is a large part of the Qinghai–Tibet Plateau and is a relative subsidence area in the Quaternary strong uplift area, with a total area of 4,247,327 hm^2^ (Figure 1). Its wetland is a typical alpine wetland on the Qinghai–Tibet Plateau’s northeast edge [28] and its main classes include marshes, meadow, and flood wetland. The elevation change in this area is 2392–5057 m; the geomorphology is characterized by wide valleys and gentle hills, and the plateau meadow and peat swamp soils are widely developed [34]. The main rivers in the area include the Yellow River and its tributaries, the Heihe and Baihe [7]. The study area is rich in animal and plant resources, with mainly subalpine meadow and swamp vegetation. Animal husbandry is the traditional pillar industry in the Zoige region, but due to the unique resources in this area, tourism has become the dominant industry in the area.

### 2.2. Data Preparation

Our data consisted of two parts: (1) for the satellite imagery analysis, Landsat imagery obtained from United States Geological Survey (USGS, https://www.usgs.gov/) [22] and terrain data with a resolution of 30 m; (2) for the landscape change driver analysis, climate and socioeconomic data from the Data Center of China Meteorological Administration (http://www.cma.gov.cn) and National Statistical Yearbooks, respectively.

#### 2.2.1. Satellite Imagery Process

Satellite imagery processing was carried out through two platforms. GEE obtained the relevant images from the integrated database through code for direct online analysis. The traditional method is to download images from the USGS data center for offline analysis. Considering cloud coverage is extensive in this area and a single image cannot satisfy the needs of the experiment, we unified the time limit for data acquisition in the growing season (July–September) [35,36], and the initial filtering defined a maximum cloud-coverage threshold of 30%. The method of multi-temporal dense time stacking [17,35,36,37,38] was used to provide a good quality image. All data, pre-processing, post-processing and methods are comprehensively described in Supplementary Materials Text S1.

#### 2.2.2. Classification Feature Collection

We extracted the classification features derived from Landsat 5/8 observation (i.e., Band1–5, 7 in Landsat5; Band2–7 in Landsat8) and Digital Elevation Model (DEM) data. Based on the surface reflectance bands, we selected and calculated the following spectral indices: (1) the Normalized Difference Vegetation Index (NDVI) [39,40], the best indicator of vegetation growth status and vegetation coverage; (2) the Normalized Difference Built-up Index (NDBI) [41], the index used to analyze the built-up area; and (3) the Normalized Difference Water Index (NDWI) [42,43] and Modified Normalized Difference Water Index (MNDWI) [44,45], which reflect the surface moisture conditions and play an important role in the extraction of wetland with abundant water information. These indices calculation formulas were summarized in Supplementary Materials Table S1. Terrain features is an important index for land cover classification in alpine areas [46]. Slope [47], aspect [48] and elevation [47] are calculated by DEM to improve classification results.

#### 2.2.3. Sample Selection

Most of the articles published in the relevant study area have been combined with the Land-Use and Land-Cover Change (LUCC) classification system, and the features were divided into seven types [49,50]. Considering the small area of farmland and building-land types in the study area, we combined them into artificial land. Six feature types remained: grassland, woodland, wetland, river, artificial land, and unused land (Supplementary Materials Table S2).

Based on extensive field investigations and unmanned aerial vehicle (UAV) data, with reference to Google Earth images from the same period, ArcGIS 10.6 was used to select and generate training samples on Landsat images. Considering the balance of samples, the number of samples is set according to the area proportion of local types [37,51]. The generated samples of 4500 points are “real” and are used for the classification and subsequent classification accuracy evaluation according to the 7:3 ratio [50,52] of training samples to test samples. We selected four kinds of accuracy evaluation indexes used in many studies to evaluate accuracy: overall accuracy, the Kappa coefficient, producer accuracy and user accuracy [52,53].

#### 2.2.4. Meteorological and Socio-Economic Data

In order to further explore the relationship between the change in the landscape pattern of Zoige alpine wetland and its influencing factors, the grey correlation analysis was used to analyze the factors affecting the change of wetland landscape pattern. We selected indicators from meteorological and socio-economic data to construct an indicator system of landscape pattern change drivers. In this study, meteorological factors (annual temperature, precipitation, and evaporation) were used to analyze the characteristics of alpine wetland area change for ZP. The temperature and precipitation data were derived from the measured data of three meteorological stations (Zoige, Hongyuan, and Maqu) on the ZP. The evaporation data were calculated from the stations’ monthly temperature and precipitation data with the following formula:

$$E=\frac{3100R}{\left(3100+1.8R^{2}\exp\left(-\frac{34.4T}{235.0+T}\right)\right)}$$

where *E* is the monthly evaporation (mm), *R* is the monthly precipitation (mm), and *T* is the monthly average temperature (°C).

**Figure 2:**
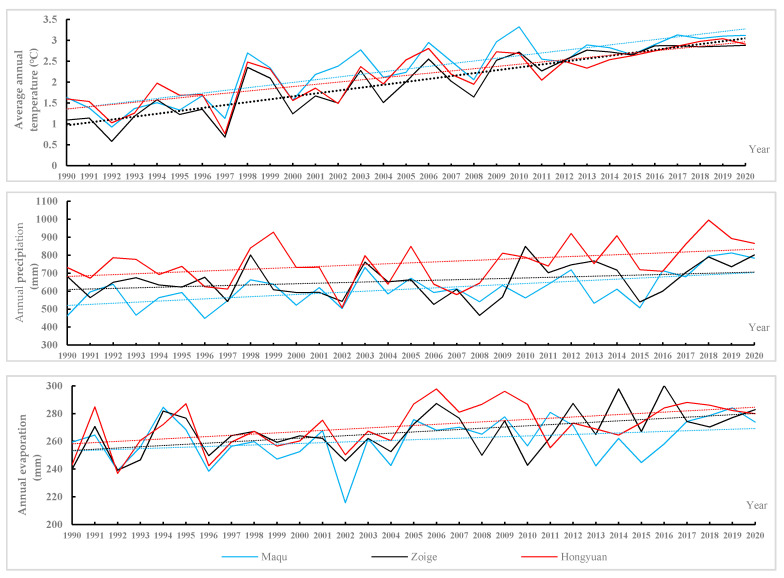
Annual temperature, precipitation, and evaporation at 3 stations, ZP, from 1990 to 2020.

Observations from three weather stations around the ZP wetland are shown in Figure 2, showing that the average annual temperature has increased significantly over 30 years, with an average temperature increase of about 1.56 °C. From 1990 to 2020, the annual precipitation on the ZP remained stable, and the trend was stable. The average annual precipitation was 682.89 mm and the average annual evaporation was 268.01 mm.

We selected the population, number of livestock, gross domestic product (GDP), primary, secondary, and tertiary industrial output value, and per capita GDP to constitute socio-economic indicators (Table 1). The missing data in 2020 were supplemented by data fitting method.

### 2.3. Methods

#### 2.3.1. Calculation Methods of Landscape Indices

According to the research needs of this study and the ecological significance of each landscape index, the selected landscape indexes include patch area index (the alpine wetland area, *WA*), patch type proportion index (the wetland percentage in total landscape, *WP*), landscape change index (*LCI*), patch number (PN), patch density (PD), landscape shape index (*LSI*), landscape diversity index (Shannon’s Diversity Index, *SHDI*) and landscape evenness index (Shannon’s Evenness Index, *SHEI*). The landscape index is calculated using Fragstats 4.2 software.

The equations below are used to calculate *WA* and *WP* (%), respectively.

$$WA=\sum_{j=1}^{n}a_{ij}[\frac{1}{10000}]\;({hm}^{2}),$$

$$WP=\frac{\sum_{j=1}^{n}a_{ij}}{A}(100)$$

where *i* is the wetland landscape; *j* as the PN, *j* = 1, ..., *n*; *a_ij_* refers to the area of the *j*th patch in the *i*th patch class, and *A* refers to the entire area of landscape.

The *WP* is also called the patch area index of the wetland landscape. It reflects elements and pattern changes in the landscape, and it is used to determine superior landscape elements. A *WP* value close to 0 indicates that the proportion of wetland in all landscape types is close to 0; in contrast, a *WP* value close to 100 indicates that wetland constitutes ~100% of the landscape.

*LCI*: defined as the absolute values of change in the land cover types that have the greatest impact on the shape of the landscape [54]. The purpose of this index is to determine the wetland *LCI* for each of the time intervals.

$$LCI_{t}=\;\frac{1}{2}\;\times \left|WP_{t+1}-WP_{t}\right|$$

where *LCI_t_* is the wetland *LCI* in each time interval; *WP_t_*_+1_ and *WP*_t_ represents *WP* during the time interval *t* + 1 and *t*.

PN refers to the total number of landscape patches; PD refers to the number of patches per hm^2^; PN and PD reflect the degree of landscape fragmentation. The greater the number of patches and landscape patch density, the greater the degree of landscape fragmentation.

*LSI*: the total boundary length of all patches in the landscape is divided by the square root of the total landscape area, and then multiplied by the correction coefficient of the square. When the patch shape in the landscape is irregular or deviates from the square, the *LSI* value increases. The formula for *LSI* is:

$$LSI=\frac{0.25E}{\sqrt{A}}$$

where *E* is the total length of all patch boundaries in the landscape.

*SHDI*: based on information theory, the *SHDI* is used to measure the complexity of the system structure. The magnitude of landscape diversity reflects the number of landscape elements and the proportion of landscape elements. For this study, *SHDI* is calculated as follows:

$$SHDI=-\overset{n}{\underset{i=1}{\sum }}P_{i}\ln\left(P_{i}\right)$$

where *P_i_* is the probability of patch type I appearing in the landscape, and *n* is the total number of patch types in the landscape. The magnitude of diversity depends on two aspects of information: one is the number of patch types (richness), and the other is the uniform distribution of patch types in the area. For a given *n*, when the proportion of all kinds of patch area is the same (that is, *P_i_* = 1/*n*), *SHDI* reaches a maximum. In general, with the increase in *SHDI*, the complexity of the landscape structure tends to increase.

*SHEI*: the evenness index, E, reflects the uneven distribution of patches in the landscape, which is usually expressed by the ratio of the diversity index to its maximum value. The formula for *SHEI* is:

$$SHEI=\frac{H}{H_{MAX}}=\frac{-\sum_{i=1}^{n}P_{i}\ln\left(P_{i}\right)}{\ln\left(n\right)}$$

where *H* is the *SHDI*, and *H_MAX_* is its maximum. When *SHEI* tends to be 1, the uniformity of landscape patch distribution also tends to be the maximum.

#### 2.3.2. Grey Relational Analysis

The grey relational analysis method [30,31] based on grey theory can determine the degree of influence of various factors on the development trend of the system, and the formula for grey correlation degree is as follows:

(1) the original data sequence includes characteristic target sequence (*x*_0_) and related factor order (*x_i_*):
  $$x_{0}=\left(x_{0}\left(1\right),x_{0}\left(2\right),\ldots ,x_{0}\left(n\right)\right)$$
  $$x_{i}=\left(x_{i}\left(1\right),x_{i}\left(2\right),\ldots ,x_{i}\left(n\right)\right)\;i=1,\ldots ,k$$
(2) calculation of correlation coefficient:
  $$\varepsilon_{i}\left(k\right)=\frac{min_{i}\left(\Delta_{i}\left(min\right)\right)+0.5max_{i}\left(\Delta_{i}\left(max\right)\right)}{\left|x_{0}\left(k\right)-x_{i}\left(k\right)\right|+0.5max_{i}\left(\Delta_{i}\left(max\right)\right)}$$
  where *ε_i_* (*k*) is the relative difference between the sequence of related factors *x_i_* and the characteristic target sequence x_0_ at time *k*, called the correlation coefficient of *x_i_* to *x*_0_ at time *k*; 0.5 is the resolution coefficient; min (Δ*i*(*min*)), the minimum difference between the two levels; max (Δ*i*(*max*)), the maximum difference between the two levels.
(3) The grey correlation calculation is:
  $$r_{i}=\frac{1}{N}\overset{n}{\underset{k=1}{\sum }}\varepsilon_{i}\left(k\right)$$
  where *r_i_*, the correlation degree between the characteristic target sequence (*x*_0_) and the related factor sequence (*x_i_*).

## 3. Results

### 3.1. Classification Results and Accuracy

**Figure 3:**
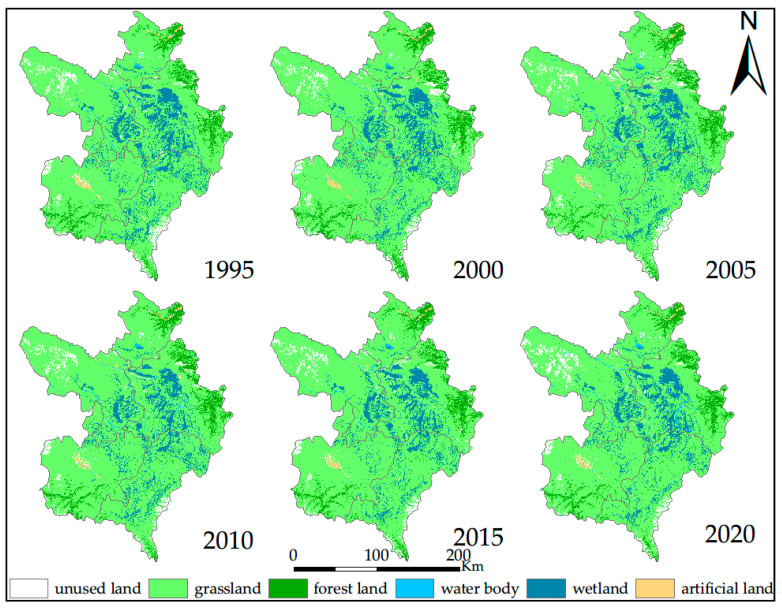
Alpine wetland distribution in ZP for different periods.

**Figure 4:**
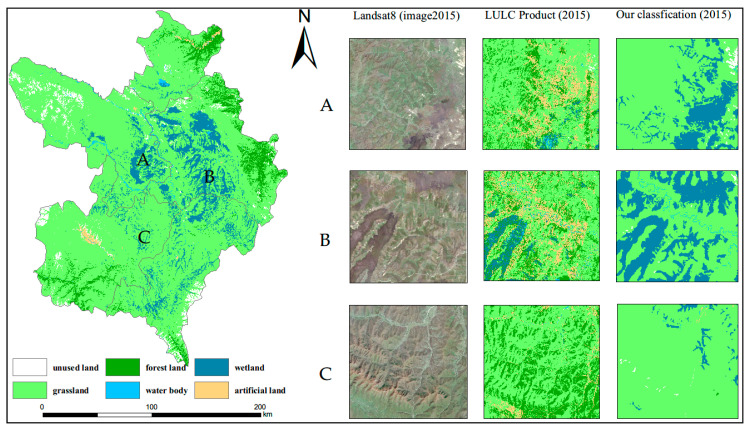
Comparison diagram of classification results (A, B and C refer to three wetland area; left refer to Landsat images in growth season; middle refers to FROM-GLC products; right refers to the classification results of our study).

The results of classification and accuracy assessment of land cover types over the six years (1995, 2000, 2005, 2010, 2015 and 2020) using three algorithms are shown in Figure 3 and Table 2. Overall, the RF and DT algorithm have a high accuracy (90.06–94.92%), while the accuracy of the SVM algorithm is relatively low (84.39–89.62%). We will not consider the SVM algorithm in the research process. In different years, the differences in the Kappa coefficient and overall accuracy are small between the RF and DT. For individual land cover classes, the classification accuracies are very good overall, and there is little difference from class to class and from year to year. Wetland classification of user accuracy and producer accuracy reached the ideal threshold range. Given that the classification accuracy of the two algorithms is very close to the overall accuracy and the Kappa coefficient, we chose the RF, which has a high accuracy and can analyze the importance of selected variables adopted by the subsequent analysis of the data. Finally, we introduced FROM-GLC version2 (2015_v1) product [14], a widely recognized global 30 m classification products (http://data.ess.tsinghua.edu.cn), to compare with the classification results in our study and further evaluate our classification results. In Figure 4, we chose three wetland areas (A, B and C) to evaluate the classification results in 2015 by visually determining the degree to which the classification results match the image of the growing season. As a whole, the spatial distribution of our classification results was consistent through visual manual inspection. Our study has a finer effect on wetland classification, while the product performs poorly in the classification of wetland in our study area.

### 3.2. Dynamics Change, from 1995 to 2020

Table 3 and Table 4 show the wetland landscape change including PN, WA, WP and LCI, based on Landsat images of the six years (1995, 2000, 2005, 2010, 2015 and 2020). From 1995 to 2005, the number of wetland patches in the study area increased from 7093 to 9410, with the biggest rise (+25.2%) occurring from 2000 to 2005. From 2005 to 2015, the number of wetland patches in the study area significantly decreased from 9410 to 5703, with the biggest drop (−32.1%) occurring from 2010 to 2015. The turning point for wetland changes was 2015; wetland area began to increase and PN increased from 5703 to 6323. The largest changes within the wetland landscape occurred in the period 2000–2005, and wetland LCI was 0.200. From 2015 to 2020, the change dynamic was the lowest, and the wetland LCI was 0.125.

In order to analyze the characteristics of wetland change, the patches were divided into 10 groups according to patch area, a frequency analysis was carried out, and the wetland in six stages was counted according to 10 areas from small to large. As can be seen from Table 5, from 1995 to 2005, the number of wetland patches smaller than 10 hm^2^ showed an obvious increasing trend (+40.1%), and the number of patches with sizes of 300~600 hm^2^ and 600~1000 hm^2^ showed an obvious decreasing trend (−29% and −25.8%, respectively), indicating some larger patches were broken into many small patches from 1995 to 2005. From 2005 to 2015, the number of wetland patches smaller than 10 hm^2^ decreased sharply (−48.9%); in fact, the overall reduction in PN was mainly concentrated in the small-patch category. From 2015 to 2020, the number of wetland patches smaller than 10 hm^2^ increased 15%, while the area distribution of other patches changed slightly.

In order to further analyze the change in wetland area, all the patches were divided into three levels (small, middle, large) according to the size of patch area (i.e., <1000 hm^2^, 1000–10,000 hm^2^, >10,000 hm^2^). The total area of wetland at all levels was counted and the ratio of the increase (or decrease) in the area in the current period compared with that in the previous period was adopted to specifically show the dynamic change of wetland area in different groups. As can be seen from Table 6, from 1995 to 2005, the total area of wetland decreased by 6.6%, the small and large levels decreased by 2.5% and 15.1%, respectively, and 1000–10,000 hm^2^ increased by 3.5%. This result, combined with other landscape parameters, shows that the decrease in wetland area in this period was mainly due to the fragmentation of larger patches into many small patches and loss of the original small patches. From 2005 to 2015, the total area of wetland decreased by 5.6%, the middle and large levels decreased by 15.3% and 4.9%, respectively, and small level increased by 2.6%. This shows that the decrease was caused by the loss of many middle patches and the decrease in large patches from the edge to the middle. From 2015 to 2020, the total area of wetland increased by 2.6%, and the small, middle, and large levels increased by 6.1%, 1.7% and 0.4%, respectively. This shows that the increase was caused by increasing in the number of smaller patches and recovery of original wetland area.

Combined with Table 3 and Table 7, the overall change trends of PN and PD of alpine wetland landscape in ZP from 1995 to 2015 are same. From 1995 to 2005, PN and PD increased, indicating that the number of patches per square kilometer increased and the degree of landscape fragmentation increased. From 2005 to 2015, PN and PD decreased, indicating the number of patches per square kilometer decreased and the degree of landscape fragmentation decreased. In 2020, PN and PD increased again, and the degree of landscape fragmentation increased.

The change in LSI is shown in Table 7. From 1995 to 2010, the LSI of wetland increased, meaning the landscape shape of wetland tended to be complicated; from 2010 to 2015, the LSI of wetland decreased, and the landscape shape of wetland tended to become homogenized. The LSI of wetland increased slightly (+1.3%) from 2015 to 2020.

The magnitude of landscape diversity depends on the number of patch types and the uniform distribution of patch types in area. As the SHDI increases, the composition of landscape structure becomes more complex. SHEI reflects the uneven distribution of patches in the landscape. The smaller the SHEI, the more uneven the patch distribution. In Table 7, the SHDI and SHEI for alpine wetland on the ZP showed a downward trend from 1995 to 2015, indicating the wetland area continued to decrease, the landscape structure of wetland tends to be simpler, the distribution of patches in area is more uneven, and the distribution of landscape types is more concentrated. From 2015 to 2020, the SHDI and SHEI showed a slight upward trend (+2.3% and +2.4%, respectively).

### 3.3. Results of Dynamic Landscape Changes

After studying the landscape pattern changes of wetland separately, we noticed the ratio of wetland to other landscapes has been changing dynamically, and we used the results of six phases of classification to make a transfer matrix in order to further explore the transformation relationship between wetland and other features. Table 8 shows that wetland is mainly transformed into grassland, with less transfer to other features; this is mainly related to the landscape distribution of local alpine areas, and the edge of wetland patches is mainly dominated by alpine grassland. With the impact of human activities and climate change, the area of wetland decreased from 1995 to 2015, and wetland continued to transform into grasslands. After 2015, however, the transformation from grassland to wetland becomes the main theme.

### 3.4. Grey Relational Analysis

The grey correlation degree between the change in wetland landscape pattern and the main climatic factors and human economic activities was calculated. Table 9 shows that annual evaporation and precipitation have the greatest influence on the area of Zoige alpine wetland, followed by the increase in population and livestock and the average annual temperature. Primary, secondary, and tertiary industries also affect the change of landscape pattern of Zoige alpine wetland to some extent.

## 4. Discussion

### 4.1. Comparison of Classification Results and Analysis of Classification Variable Importance

In Table 2, we compare the classification accuracy of the three machine learning algorithms in this area. The results show that RF and DT have higher user accuracy, producer accuracy, overall accuracy, and Kappa coefficient, and their performance is better than that of the SVM. Research showed that the classification result of the SVM is ideal in the case of few feature parameters [20]. Based on this, we think the poor performance of the SVM is related to the number of characteristic variables in this study. In addition, we notice there is little difference in accuracy between the RF and the DT. Based on the literature, we know the DT has certain shortcomings compared with the RF [55,56]. This is mainly reflected in the higher requirements for the selection of feature parameters before classification, and the selection and understanding of feature parameters directly determine the classification accuracy [56,57]. In this experiment, the small difference in accuracy between the two algorithms shows that our selected features basically meet the requirements of classification.

RF is a kind of non-parametric classifier [58] which does not need to consider statistical distribution. Because it may be less sensitive to noise and more efficient in processing highly dimensional data [56], it has become the most popular classifier at present. However, the most important point and the reason for our choice of this algorithm is that it can analyze the importance of feature variables [59] and reduce data redundancy and processing workload while improving the accuracy of the model. We can make full use of this performance to do more in-depth research, such as increasing the number of feature parameters and trying many parameter combinations to obtain the best classification method. In addition, we note that some scholars will give different numbers of characteristic parameters to the two machine learning algorithms (RF and SVM) when classifying [20]. This method avoids the weakness, in that the SVM is not suitable for dealing with high-dimensional data. Nevertheless, it means that the SVM cannot give full play to its advantages. The best solution is to use the RF. In other words, the efficiency of feature variables is maximized by the RF, and these selected feature parameters are used to make the SVM have a better performance.

We calculated the average of the importance score of the six periods for each characteristic variable and sorted them from high to low (Supplementary Materials Table S3). The higher the important score in the image, the greater the influence of this variable on the classification results. In Table 3, we can see that variables such as elevation, MNDWI, slope, NDVI and NDBI have higher importance scores. The spatial distribution of alpine area is greatly affected by topography and altitude, so the introduction of terrain factors (i.e., elevation and slope) as an important part of characteristic parameters can effectively classify the land types in this area [46,60]. MNDWI can maximize the inhibition of vegetation information and highlight water bodies [44], which can distinguish part of the alpine grassland and meadow wetland. NDVI is the best indicator of vegetation coverage and can acquisition a good distinction between vegetation area and non-vegetation area [39]. NDBI is the index used to analyze the built-up area [41]. However, since the main cover types of the study area are alpine grassland and alpine wetland, the importance of this index is less than that of MNDWI and NDVI. The importance of several bands in the image output of Landsat (i.e., Blue, Green, Red, NIR, SWIR1 and SWIR2) is at a medium level, mainly because the previous indexes (i.e., NDVI, MNDWI and NDBI) is calculated by these bands and contains a large amount of original information. MNDWI and NDWI was widely used [17,61] to detect water bodies. However, NDWI does not perform as well as MNDWI in this study, which may be due to the combination of the MNDWI and NDVI, which can reduce the impact of vegetation on water monitoring and easy to have a better performance in alpine wetland with abundant vegetation information and water body information.

### 4.2. Analysis of Landscape Dynamics and Drivers of Wetland Changes

From 1995 to 2015, the alpine wetland on the ZP continued to degrade, and the area decreased year by year. The area of wetland increased from 2015 to 2020 but is still lower than during the 1990s. Combined with frequency and landscape dynamic analysis, the decrease in wetland area from 1995 to 2005 was mainly due to the fragmentation of larger patches into many small patches and the loss of the original small patches. The decrease in wetland area from 2005 to 2015 was due to the loss of many middle patches and the decrease in large patches from the edge to the middle. The increase in wetland area from 2015 to 2020 was due to the increase in the number of smaller patches and the expansion of part of the original patch area.

From 1995 to 2015, the temperature in ZP increased rapidly and precipitation remained stable; this led to a substantial increase in evaporation and insufficient rainfall recharge, and it became difficult for wetland to maintain water levels. In addition, the overall surface water resources in ZP showed a fluctuating downward trend over the past 30 years [62], which caused the area of alpine wetland in ZP to decrease continuously from 1995 to 2015. The area of alpine wetland in ZP decreased the most from 2000 to 2005, while the temperature and evaporation increased faster from 2000 to 2005 than during other periods. From 2015 to 2020, the temperature in ZP continued to increase rapidly. The main reason for the increase in wetland area from 2015 to 2020 was the change in climate factors and the decline of livestock carrying capacity. The grey correlation degree further shows that precipitation and evaporation are the main factors leading to the change of landscape pattern of ZP wetland.

We note from Table 3 the area of wetland degradation in 2005 to 2015 decreased compared to 1995 to 2005. Combined with Table 1, this shows that the industrial structure of ZP gradually tends to be stable. This is mainly due to the implementation of the Grassland Law in 2003. The National Ecological Function Regionalization issued by the Ministry of Environment in 2008 divides the ZP into important areas for water conservation. The implementation of the Grassland Law and the release of the National Ecological Function Regionalization have promoted the formulation of a series of ecological protection measures, such as the implementation of the ecological migration project and measures to adjust the policy industrial structure and develop ecotourism. The implementation of these policies has played a certain role in alleviating the degradation of wetland. However, due to the influence of evaporation, annual precipitation, population, and livestock (population and livestock rose from 2005 to 2010, with a downward trend from 2010 to 2015, but population still increased rapidly overall), the situation of wetland degradation from 2005 to 2015 is still grim.

In Table 1, we can see that the number of livestock in ZP continues to increase, the economic growth of the region has accelerated since 2005, and the proportion of tertiary industry has increased. The development of tourism in this region has promoted the development of local transportation, accommodation, catering, and other service industries, but the rising number of tourists has led to excessive pressure on the population of the region. This will inevitably lead to a sharp increase in the resource consumption in the region, and to the plunder and destruction of wetland resources. Related studies [63,64] have shown the livestock numbers on the ZP have seriously exceeded the actual livestock carrying capacity of the grassland. The average overloading rates of Zoige, Hongyuan, and Aba in 2000 and 2007 were 92% and 128%, respectively, with 72% in Maqu County and 71.5% in Luqu County in 2007. Moreover, the phenomenon of trenching and drainage still exists. There are 17 canals in marshes such as Xiaman Township and Heihe pasture, with a total length of 50.5 km; this turns the swamps of Heihe into grazing land, resulting in wetland degradation. Although the wetland has been restored by measures such as filling and blocking water in more than 20 places since the establishment of the protected area in 2010, due to the decline in groundwater level, the original channel is still draining a large amount of water. Although the industrial structure in ZP tends to be rationalized, it is evident that primary, secondary, and tertiary industries have little influence on the wetland landscape pattern, as seen in Table 9. The adjustment effect of industrial structure is not significant, and the conflict between supply and demand for water resources in this area is still serious, which is not conducive to the protection of alpine wetland. From 2015 to 2020, the population of ZP tended to be stable, annual precipitation increased rapidly, evaporation decreased relatively, and livestock decreased by 238,300 head compared with 2015. In 2019, the local government built a new Zoige wetland science education base to strengthen wetland protection, while a greater emphasis on rational tourism development provided new employment opportunities for the surplus labor force. These measures promote the adjustment of land use and rural industrial structure, resulting in a significant increase in ecological benefits; the interaction of the above factors resulted in a corresponding increase in wetland area by 2020.

Landscape change analysis is an important part of wetland research. Our study on the dynamic change of wetland was all calculated and analyzed by Fragstats4.2 and we also note scholars in relevant studies [65] used the Landscape Fragmentation Tool v2.0 (LFT) created by the University of Connecticut’s Centre for Land Use Education and Research (CLEAR) for producing and visualizing fragmentation metrics. In their study, urban wetland in Khushalsar was taken as the object of study [65]. Four types of landscape were analyzed and revealed using LFT, and the causes of wetland degradation were analyzed accordingly.

### 4.3. Limitations of the Current Study and Platforms Selection

In order to monitor wetland changes on a long time series scale, some high-resolution images (including the Sentinel imagery) cannot be used in early wetland monitoring. Another dilemma is that the images in this area have been subject to the influence of cloud cover all the year round, and we have adopted the method of multitemporal dense time stacking. Although this method basically removes the influence of cloud cover on the experiment, because the pixel is in an inconsistent time, it also adds uncertainty to the classification process and results. Fortunately, the development of active remote sensing has brought us to a new dawn of understanding, and exploring the integration of optical and radar data to solve the impact of cloud cover on regional monitoring is another development trend in this field [66,67,68] at present and beyond.

There are always challenges in traditional image analysis, including image downloading and storage, data processing, and preprocessing [69]. Therefore, previous studies have also focused on small areas. In recent years, with the emergence of GEE, great changes have taken place in the research field and depth. GEE brings us many advantages [21]; for example, it allows geospatial analysis to be carried out over the whole space–time continuum, bringing unprecedented processing efficiency. At present, GEE is recognized as a tool with immense computational power. It provides two programming interfaces: JavaScript and Python. Many researchers are constantly improving its functions, and the future GEE will be more powerful. The time and effort spent by the traditional method in small area image processing is still acceptable and it has classification accuracy equal to or even higher than that of GEE, but the better choice is definitely the GEE platform.

## 5. Conclusions

In this study, the dense time stacking of multi-temporal method and growing season Landsat images are used to monitor the dynamic changes in ZP. Through the landscape analysis of three scales, the change direction of the landscape is obtained, and the driving force is analyzed by grey correlation degree.

Our results show the ZP wetland is in a state of degradation during the period from 1995 to 2015, and the wetland area begins to increase after 2015. The degree of fragmentation of the wetland landscape increased at first and then decreased, and the landscape shape gradually shifted from complexity to homogenization. Landscape diversity continues to decrease, the distribution of large wetland patches is more concentrated, and the area distribution is more uneven. Climate change may be the main factor affecting the proportion of wetland landscape in this area. The RF has the best overall classification effect, and GEE will gradually become an indispensable cloud processing tool.

The study shows that in the future utilization and protection of alpine wetland, we should strengthen reasonable policy guidance, improve the comprehensive management level of wetland reserves, scientifically protect wetland resources, improve the industrial structure of wetland areas, and restrict industrial development. We should promote the development of modern water-saving and energy-saving industries and prevent the destruction of wetland resources by traditional industrial water and land uses.

## Figures and Tables

**Table 1:** Socioeconomic data for ZP, from 1995 to 2020.

Year	Gross Domestic Product (10^4^ Yuan)	I Primary Industry (10^4^ Yuan)	II Secondary Industry (10^4^ Yuan)	III Tertiary Industry (10^4^ Yuan)	Industrial Structure %	Population (10^4^)	Number of Livestock (10^4^)	Per Capita GDP (10^4^ Yuan)
1995	62,420	42,268	9881	10,271	68:16:16	21.5	203.42	0.3054
2000	87,261	49,939	17,953	19,369	57:21:22	22.65	295.38	0.402
2005	156,655	70,133	38,493	48,029	45:24:31	25.09	336.4	0.6481
2010	335,526	133,604	82,011	119,911	40:24:36	27.84	387.22	1.2753
2015	587,772	214,098	129,436	244,238	36:22:42	29.01	355.08	2.0902
2020	798,323	316,934	210,757	270,632	40:24:36	30.26	320.52	2.6913

**Table 2:** Accuracy results for land cover types for 6 years with 3 algorithms.

Types	Platform-Classifier	6 Periods											
		1995		2000		2005		2010		2015		2020	
		UA	PA	UA	PA	UA	PA	UA	PA	UA	PA	UA	PA
Grassland	RF-GEE	89.74%	97.36%	90.38%	96.64%	88.48%	96.75%	89.45%	93.15%	93.81%	96.72%	90.26%	93.83%
	SVM-GEE	86.86%	96.04%	87.57%	90.84%	85.71%	96.34%	80.59%	88.36%	90.20%	88.89%	88.36%	88.51%
	DT-Envi	92.60%	95.89%	88.68%	96.04%	89.45%	96.04%	89.06%	96.13%	92.76%	96.74%	88.39%	92.90%
Wetland	RF-GEE	94.23%	89.42%	89.12%	86.94%	93.25%	85.14%	87.79%	85.50%	94.92%	94.92%	89.96%	87.59%
	SVM-GEE	88.85%	90.15%	88.00%	89.80%	90.66%	84.42%	91.71%	83.12%	93.33%	91.30%	82.87%	89.10%
	DT-Envi	89.24%	91.43%	91.79%	89.78%	92.80%	86.25%	91.51%	89.86%	92.51%	92.86%	92.66%	86.96%
Forest	RF-GEE	97.37%	95.36%	98.21%	97.06%	96.24%	94.21%	96.65%	97.74%	96.59%	96.05%	96.02%	95.54%
	SVM-GEE	98.88%	90.72%	96.73%	87.06%	97.31%	95.26%	94.48%	90.96%	88.37%	85.88%	95.79%	90.10%
	DT-Envi	97.63%	97.06%	97.93%	97.42%	94.94%	96.02%	98.40%	96.86%	96.45%	95.48%	93.18%	92.66%
Water	RF-GEE	92.45%	79.67%	91.18%	78.81%	90.35%	83.74%	93.20%	82.76%	98.13%	88.24%	93.50%	87.12%
	SVM-GEE	84.40%	74.80%	76.61%	80.50%	89.38%	82.11%	84.72%	78.74%	93.46%	84.03%	86.07%	79.55%
	DT-Envi	97.85%	77.12%	95.00%	77.24%	96.08%	84.48%	93.14%	77.24%	92.62%	85.61%	93.97%	91.60%
Artificial land	RF-GEE	89.76%	89.06%	91.82%	90.18%	93.40%	86.84%	94.34%	95.26%	97.89%	93.94%	94.12%	89.60%
	SVM-GEE	87.25%	69.53%	92.40%	65.18%	85.71%	84.21%	73.33%	69.18%	88.76%	79.80%	93.75%	72.00%
	DT-Envi	88.24%	93.75%	88.98%	82.03%	94.06%	90.48%	89.38%	88.60%	96.52%	88.80%	93.33%	84.85%
Unused land	RF-GEE	96.43%	91.53%	95.95%	89.31%	93.83%	93.25%	89.36%	85.71%	92.99%	92.99%	88.36%	88.36%
	SVM-GEE	91.91%	89.83%	75.28%	84.28%	96.35%	80.98%	84.80%	88.96%	71.72%	90.45%	75.80%	87.83%
	DT-Envi	96.08%	92.45%	94.80%	92.66%	94.25%	90.34%	93.33%	88.05%	94.02%	92.02%	83.75%	84.81%
Overall accuracy	RF-GEE	92.57%		91.93%		91.61%		90.72%		94.92%		91.32%	
	SVM-GEE	89.20%		86.33%		89.62%		84.39%		88.09%		86.57%	
	DT-Envi	92.98%		91.73%		92.10%		91.65%		93.64%		90.06%	
Kappa coefficient	RF-GEE	90.32%		89.30%		89.11%		87.65%		93.27%		88.63%	
	SVM-GEE	85.86%		81.94%		86.49%		81.25%		84.33%		82.47%	
	DT-Envi	90.73%		89.22%		89.42%		89.17%		91.65%		86.81%	

**Table 3:** Patch number (PN), wetland area (WA), and wetland percentage (WP).

Year	PN/n	WA/hm^2^	WP/%
1995	7093 (*)	450,642.87 (*)	10.61 (*)
2000	7515 (+5.9%)	437,998.77 (−2.8%)	10.31 (−2.8%)
2005	9410 (+25.2%)	420,869.7 (−3.9%)	9.91 (−3.9%)
2010	8403 (−11.7%)	409,675.86 (−2.6%)	9.65 (−2.6%)
2015	5703 (−32.1%)	397,207.44 (−3.1%)	9.35 (−3.1%)
2020	6323 (+10.9%)	407,560.05 (+2.7%)	9.60 (+2.7%)

Note: The (*) indicate the initial parameters size; the (…%) indicate the percentage change at time *t* + 1 relative to time *t*.

**Table 4:** Landscape change index (LCI) from 1995 to 2020.

Time Interval	Wetland LCI
1995–2000	0.150
2000–2005	0.200
2005–2010	0.130
2010–2015	0.150
2015–2020	0.125

**Table 5:** PN in different areas of alpine wetland, ZP, from 1995 to 2020.

	Number of Patches/n	1995	2000	2005	2010	2015	2020
Area/hm^2^							
<10		5579 (*)	5773 (+3.5%)	7817 (+35.4%)	6634 (−15.1%)	4000 (−39.7%)	4601 (+15.0%)
10~25		580 (*)	719 (+24.0%)	622 (−13.5%)	693 (+11.4%)	680 (−1.9%)	701 (+3.1%)
25~50		361 (*)	372 (+3.0%)	366 (−1.6%)	376 (+2.7%)	387 (+2.9%)	363 (−6.2%)
50~100		221 (*)	271 (+22.6%)	251 (−7.4%)	290 (+15.5%)	293 (+1.0%)	288 (−1.7%)
100~300		195 (*)	233 (+19.5%)	224 (−3.9%)	233 (+4.0%)	211 (−9.4%)	234 (+10.9%)
300~600		73 (*)	60 (−17.8%)	54 (−10%)	77 (+42.6%)	59 (−23.4%)	57 (−3.4%)
600~1000		31 (*)	40 (+29.0%)	23 (−42.5%)	39 (+69.6%)	24 (−38.5%)	26 (+8.3%)
1000~3000		34 (*)	32 (−5.9%)	33 (+3.1%)	42 (+27.3%)	32 (−23.8%)	35 (+9.4%)
3000~10,000		12 (*)	9 (−25.0%)	13 (+44.4%)	14 (+7.7%)	10 (−28.6%)	11 (+10%)
>10,000		7 (*)	6 (−14.3%)	7 (+16.7%)	5 (−28.6%)	7 (+40%)	7 (0)

Note: The (*) indicate the initial parameters size; the (…%) indicate the percentage change at time *t* + 1 relative to time *t*.

**Table 6:** The alpine wetland in the study area consisted of different patches, from 1995 to 2020.

	1995	2000	2005	2010	2015	2020
<1000 hm^2^ (small)	131,056.67 (*)	145,567.17 (+11.0%)	127,784.52 (−12.2%)	156,258.81 (+22.3%)	131,129.28 (−16.1%)	139,128.06 (+6.1%)
1000–10,000 hm^2^ (middle)	116,713.17 (*)	101,714.04 (−12.9%)	120,763.89 (+18.7%)	140,681.34 (+16.5%)	102,240.45 (−27.3%)	103,977.55 (+1.7%)
>10,000 hm^2^ (large)	202,873 (*)	190,717.5 (−6%)	172,321.3 (−16%)	112,735.7 (−34.6%)	163,837.89 (+45.3%)	164,454.4 (+0.4%)
all	450,642.87 (*)	437,998.77 (−2.8%)	420,869.7 (−3.9%)	409,675.86 (−2.7%)	397,207.44 (−3.1%)	407,560.05 (+2.6%)

Note: The (*) indicate the initial parameters size; the (…%) indicate the percentage change at time *t* + 1 relative to time *t*.

**Table 7:** Patch density (PD), landscape shape index (LSI), Shannon’s Diversity Index (SHDI) and Shannon’s Evenness Index (SHEI).

Year	PD	LSI	SHDI	SHEI
1995	0.1667 (*)	93.3986 (*)	0.3379 (*)	0.4875 (*)
2000	0.1767 (+6.0%)	97.421 (+4.3%)	0.3316 (−1.9%)	0.4783 (−1.9%)
2005	0.2212 (+25.2%)	98.4492 (+1.1%)	0.3227 (−2.7%)	0.4656 (−2.7%)
2010	0.1975 (−11.7%)	107.8013 (+9.5%)	0.3169 (−1.8%)	0.4572 (−1.8%)
2015	0.1341 (−32.1%)	88.8969 (−17.5%)	0.3103 (−2.1%)	0.4476 (−2.1%)
2020	0.1549 (+15.5%)	90.0597 (+1.3%)	0.3177 (+2.3%)	0.4584 (+2.4%)

Note: The (*) indicate the initial landscape parameters size; the (…%) indicate the percentage change at time *t* + 1 relative to time *t*.

**Table 8:** Transfer matrix of six phases between wetland and other features.

Transfer Type/Time	Change Area (hm^2^)				
	1995–2000	2000–2005	2005–2010	2010–2015	2015–2020
Wetland to grassland	77,471.8	107,182.2	99,318	105,216.8	88,084.9
Grassland to wetland	65,066.7	89,893.4	89,119.9	92,147.1	97,577.4
Wetland to others	80,619.2	109,085.8	101,734.9	106,992.4	89,614.4

**Table 9:** Correlation degree and correlation sequence of wetland area, with influencing factors.

Influencing Factor	Annual Evaporation	Annual Precipitation	Total Population	Large Livestock Inventory	Average Annual Temperature	Primary Industry	Secondary Industry	Tertiary Industry
Correlation degree	0.953	0.875	0.851	0.837	0.758	0.607	0.572	0.548
Sort	1	2	3	4	5	6	7	8

## Supplementary Materials

The following are available online at https://www.mdpi.com/1424-8220/20/24/7315/s1, Table S1: Several popular indices and their formulas; Table S2: Definition of land use classes of classification scheme adopted in this study; Table S3: The order of importance of feature variables in classification; Table S4: Images comprising each study year and the previous and subsequent years composite; Text S1: all data, pre-processing, post-processing, methods.
