# Supplementary material for: Monitoring and Landscape Dynamic Analysis of Alpine Wetland Area Based on Multiple Algorithms: A Case Study of Zoige Plateau
Source: Sensors (Basel). 2020 Dec 19;20(24):7315. doi: 10.3390/s20247315 (PMC7766642; doi:10.3390/s20247315)
Supplement: Supplementary file 1 [file sensors-20-07315-s001.pdf]

Supplementary Material

Monitoring and Landscape Dynamic Analysis of Alpine Wetland Area Based on Multiple Algorithms: A Case Study of Zoige Plateau

Table S1. Several popular indices and their formulas

Indices	Formulas
NDWI	(GREEN-NIR)/(GREEN+NIR)
NDVI	(NIR-RED)/(NIR+RED)
NDBI	(SWIR1-NIR)/(SWIR1+NIR)
MNDWI	(GREEN-SWIR1)/(GREEN+SWIR1)

Table S2. Definition of land use classes of classification scheme adopted in this study.

Types	Value	Description
Wetland	1	marshes, meadow, and flood wetlands.
Grassland	2	Dominated by herbaceous annuals (<2 m).
Forest land	3	Dominated by trees (canopy>2 m). Tree cover >60%.
Water body	4	Rivers and lakes.
Artificial land	5	At least 30% impervious surface area.<br>At least 60% of area is cultivated cropland.
Unused land	6	Sandy, saline, bare, desert, snow, and ice.

Table S3. The order of importance of feature variables in classification

variables	order	variables	order	variables	order	variables	order
Elevation	1	NDBI	5	Blue	9	Aspect	13
MNDWI	2	SWIR 1	6	NIR	10		
Slope	3	Green	7	NDWI	11		
NDVI	4	Red	8	SWIR 2	13		

Table S4. Images comprising each study year and the previous and subsequent years composite.

Years	Months	Paths	Rows	No. of Images(cloud<30%)
1994-1996	7-9	130-132	36-38	27
1999-2001	7-9	130-132	36-38	35
2004-2006	7-9	130-132	36-38	25
2009-2011	7-9	130-132	36-38	26
2014-2016	7-9	130-132	36-38	35
2018-2020	7-9	130-132	36-38	35

Text S1. All data, pre-processing, post-processing, methods

Random forest (RF) and support vector machine (SVM) supervision classfication algorithms are used on Google Earth Engine cloud processing platform, while decision tree (DT) on the traditional (ENVI) platform. In terms of classification indices, training sample, post-processing, etc., the two platforms are consistent.

GEE platform

All processing of Landsat TM and OLI data was conducted on the GEE platform (https://earthengine.google.com). The image processing mainly included the following steps:

Selecting all image data ("LANDSAT/LC05/C01/T1"and"LANDSAT/LC08/C01/T1") of the growth season (July–September) for each study year (1995, 2000, 2005, 2010(Landsat5); 2015, 2020(Landsat8)). The images before and after the years (defines a maximum cloud-coverage threshold of 30%, Supplementary Materials Table S4) were used to replace and supplement the images covered by clouds and fogand.

The function (ee.Algorithms.Landsat.simpleCloudScore and median ee.Reducer) was used to generate a single image from the image from the image collection. Calculate the selected classfication characteristic parameters according to the formula (Supplementary Materials Table S1) and was combined in the image obtained previously. Through the code (https://developers.google.com/earth-engine/classification) to complete the pre-processing. The results of the classification sample selection were uploaded to the personal asset library on GEE to join the training and validation sessions later.

The ee.Classifier.smileRandomForest (RF) and ee.Classifier.libsvm (SVM) was applied in the GEE platform to obtain the classification maps. The results of GEE classification were exported from the platform to the local computer and software Fratstats was used for landscape dynamic analysis.

Traditional platform

All processing of Landsat TM and OLI data was conducted on the ENVI platform. The image processing mainly included the following steps:

We downloaded remote sensing images of landsat5 (TM) and landsat8 (OLI) from USGS data center (earthexplorer.usgs.gov). The row numbers in the WRS-2 reference system are 130/037, 131/036, 131/037, 131/ 038, 132/036 and 132/037 respectively. The DEM data —

ASTERDEM V2 was downloaded from the geospatial data cloud (http://www.gscloud.cn/).

In the ENVI5.3 version software, the seamless mosaic tool is selected to splice the six images covering the study area. Histogram matching and edge feathering are carried out during stitching to reduce or eliminate the color difference of adjacent images as much as possible to ensure the quality of image mosaic. By using the image mosaic module to get the remote sensing images of the study area. All remote sensing images are uniformly used WGS_1984 ellipsoid and UTM projection.

The decision tree algorithm is selected for classification to obtain the classification results and the subsequent landscape dynamic analysis is carried out by Fratstats software.
